# Clinical and Microbiological Characteristics of Sterile-Site *Saccharomyces cerevisiae* Infections in a Tertiary-Care Centre

**DOI:** 10.1007/s11046-026-01080-7

**Published:** 2026-05-14

**Authors:** Andrea Harmath, Lilla Tünde Vass, Ágnes Jakab, Zoltán Tóth, Aliz Bozó, László Majoros, Walter P. Pfliegler, Renátó Kovács

**Affiliations:** 1 Department of Medical Microbiology, Faculty of Medicine, University of Debrecen, Nagyerdei Krt. 98, Debrecen, 4032 Hungary; 2 Doctoral School of Pharmaceutical Sciences, University of Debrecen, Nagyerdei Krt. 98, Debrecen, 4032 Hungary; 3 Medical Microbiology, Clinical Centre, Faculty of Medicine, University of Debrecen, Nagyerdei Krt. 98, Debrecen, 4032 Hungary; 4 Department of Molecular Biotechnology and Microbiology, Faculty of Science and Technology, University of Debrecen, Egyetem Tér 1, Debrecen, 4032 Hungary; 5 HUN-REN-UD Fungal Stress Biology Research Group, Egyetem Tér 1, Debrecen, 4032 Hungary

**Keywords:** *Saccharomyces cerevisiae*, Probiotics, Fungemia, Intensive care unit, Epidemiology

## Abstract

*Saccharomyces cerevisiae* has increasingly been recognized as an opportunistic pathogen. Sterile-site infections remain rare but are associated with substantial morbidity and mortality, especially in critically ill patients. This study aimed to characterize the epidemiological, clinical, and microbiological characteristics of sterile-site *S. cerevisiae* infections. We performed a retrospective analysis of all patients with *S. cerevisiae* isolated from sterile body sites between January 2019 and December 2025. Demographic data, underlying conditions, probiotic exposure, antifungal therapy, and outcomes were collected, and antifungal susceptibility testing was performed. Among 196 patients with *S. cerevisiae* isolates, 30 (15%) had sterile-site infections. The mean age was 47.2 years, and 23% were paediatric cases. Bloodstream infections were the most common source (n = 7, 23%), followed by intra-abdominal abscess aspirates and drained intra-abdominal wounds (each n = 5, 17%). Intensive care unit (ICU) admission occurred in 33%, prior gastrointestinal surgery in 63%, central venous catheter usage in all fatal cases, and prior probiotic exposure in 30%. Overall, 30-day mortality was 27%. In multivariable analysis, increasing age (adjusted OR 1.11 per year, 95% CI 1.02–1.37; *p* = 0.008) and ICU admission (adjusted OR 10.09, 95% CI 1.23–259.6; *p* = 0.03) were independent predictors of mortality. Fluconazole showed variable activity, whereas amphotericin B and echinocandins demonstrated low minimal inhibitory concentrations. Rare sterile-site *S. cerevisiae* infections are associated with high mortality related to host factors such as age and critical illness, supporting cautious probiotic use and first-line amphotericin B or echinocandins.

## Introduction

*Saccharomyces cerevisiae*, commonly known as baker’s or brewer’s yeast, is widely used in everyday life and has long been used as a probiotic microorganism in various healthcare settings [1, 2]. For decades, it was considered a generally harmless commensal yeast, capable of transient colonization of the skin, oral cavity, and the respiratory and gastrointestinal tracts of healthy individuals [3]. In recent decades, however, *S. cerevisiae* has increasingly been recognized as an opportunistic pathogen, with the potential to cause invasive infections, particularly among immunocompromised and critically ill patients, most commonly documented through isolation from normally sterile body sites [4, 5].

The mid-twentieth century introduction of *S. cerevisiae* var. *‘boulardii’* or simply *S. ‘boulardii’*—a subtype of *S. cerevisiae*—as a therapeutic probiotic, followed by its widespread use for the prevention and treatment of antibiotic-associated diarrhoea, including *Clostridioides difficile* infection, may have influenced the epidemiology of *S. cerevisiae* infections [6]. The European Confederation of Medical Mycology’s (ECMM) global guideline on rare yeast infections highlights *S. cerevisiae* as an emerging fungal pathogen, emphasizing the need for clinical vigilance comparable to that applied to other uncommon yeasts, such as *Kodamaea*, *Saprochaete*, and *Magnusiomyces* species [7].

Predisposing factors for invasive *S. cerevisiae* infection substantially overlap with those associated with candidaemia, including intensive care unit (ICU) admission, total parenteral nutrition, central venous catheterization, gastrointestinal surgery, and neutropenia [4, 5]. Transmission appears to be multifactorial, involving endogenous translocation from the gastrointestinal tract as well as colonization of central venous catheters [4, 5, 8–10]. In addition, environmental sources or person-to-person transmission have also been reported [11, 12]. Most published data consist of single case reports or small case series; consequently, the true incidence of invasive *Saccharomyces* spp. infections documented from normally sterile body sites in hospital settings remains poorly defined. Available estimates suggest an incidence ranging from 0.1 to 5% [4, 5, 11, 12].

Clinically, invasive disease most frequently manifests as fungaemia, endocarditis, or peritonitis, although involvement of other organ systems has also been described [4, 5]. Management typically includes discontinuation of probiotic yeast therapy when applicable, removal of indwelling intravascular devices, and initiation of systemic antifungal treatment [13]. Current treatment recommendations are based on limited evidence, partly because standardized clinical breakpoints and epidemiological cut-off values are not well established. However, ECMM/International Society for Human and Animal Mycology (ISHAM)/American Society for Microbiology (ASM) guidelines recommend amphotericin B as first-line therapy and echinocandins as an alternative for severe invasive *S. cerevisiae* infections. [7, 14, 15].

To date, only a limited number of studies have systematically examined invasive *S. cerevisiae* infections, leaving important epidemiological and clinical aspects insufficiently characterized. Against this background, the present study aims to address this knowledge gap by providing the first comprehensive assessment of *S. cerevisiae* infections documented from normally sterile sites at our university tertiary-care centre. By delineating their epidemiological patterns and clinical features, this work offers essential evidence to support a more complete understanding of this underrecognized pathogen.

## Materials and Methods

### Patients and Definitions

This study was conducted at the Clinical Centre of the University of Debrecen (Great Forest Campus), a tertiary-care institution with more than 1,700 beds, in Hungary. All patients with at least one episode of *S. cerevisiae* isolation from normally sterile body sites between January 2019 and December 2025 were assessed (n = 30), and all identified cases fulfilled the predefined criteria for sterile-site infection and were therefore included in the final analysis. Sterile specimens were defined as samples obtained from body sites that are normally free of microorganisms, including blood, cerebrospinal fluid, pleural, pericardial, peritoneal, synovial and amniotic fluids. In addition, bile, bone marrow, vitreous or aqueous humour, abscess aspirates, intraoperatively obtained deep tissue or organ specimens (e.g. liver, kidney, lymph nodes), bone samples, and surgically removed heart valves or vascular grafts were considered sterile. For specimens originating from deep wounds, burn wounds, abscesses or intra-abdominal compartments, only intraoperatively collected specimens or material obtained by percutaneous drainage under sterile conditions were involved. Classification as sterile-site infection required compatible clinical findings (e.g. peritonitis, abscess formation, sepsis) in addition to microbiological isolation. Radiological imaging and histological examination were not uniformly available in all cases and therefore were not required as mandatory inclusion criteria. In patients with multiple episodes of invasive *S. cerevisiae* infection, only the first episode was included in the detailed analysis. Data on patient demographics, underlying medical conditions, and details of antimicrobial therapy were retrieved retrospectively from medical records. Severe neutropenia was defined as an absolute neutrophil count < 500 cells/µL at the time of isolation of *S. cerevisiae*. Length of hospital stay was calculated from hospital admission to discharge or death. Gastrointestinal tract surgery referred to any abdominal surgical intervention involving opening of the gastrointestinal lumen within 30 days prior to isolation of *S. cerevisiae*. Clinical outcomes were assessed from the first *S. cerevisiae* positive episode until 30 days thereafter or death.

### *Saccharomyces cerevisiae* Identification and Susceptibility Testing

*S. cerevisiae* isolates were identified using matrix-assisted laser desorption ionisation/time-of-flight (MALDI/TOF) analysis as described previously [16, 17]. Briefly, identification was performed using a Bruker Biotyper Microflex LT instrument (Bruker Corporation, Billerica, MA, USA) following standard formic acid extraction and α-cyano-4-hydroxycinnamic acid treatment. Log(score) values ≥ 2.0 were considered acceptable for species-level identification. After the identification, all isolates were stored in liquid bouillon with 15% glycerine at − 20 °C until they were tested. All stored *S. cerevisiae* isolates were enriched in liquid Sabouraud dextrose agar (SDA) and plated on SDA for antifungal-susceptibility testing. The susceptibility of different isolates to fluconazole, amphotericin B, anidulafungin, caspofungin, and micafungin (all antifungals were from Merck, Budapest, Hungary) was determined using the broth microdilution method in RPMI-1640 (with L-glutamine and without bicarbonate, pH 7.0 with [3-(N- morpholino) propanesulfonic acid] buffer; Merck, Budapest, Hungary), using the Clinical and Laboratory Standards Institute (CLSI) standard M27-A3 guideline [18]. The working concentrations of the antifungals tested were 0.064–32 µg/mL, 0.015–8 µg/mL, and 0.004–2 µg/mL for fluconazole, amphotericin B, and the three echinocandins, respectively. As CLSI clinical breakpoints are not currently available for all antifungal agents, the CLSI M57/Sed4 document was used to interpret the minimum inhibitory concentrations (MICs), and isolates were categorized as wild type or non–wild type based on the corresponding ECVs [19]. When a MIC value exceeded the highest concentration tested (off-scale), one dilution above the upper limit was assigned for analytical purposes, as commonly applied in MIC distribution studies. All isolates were tested in three independent experiments, and the median MIC values were used for subsequent analyses to ensure reproducibility. Quality control was performed in each experiment using *Candida parapsilosis* ATCC 22019 and *C. krusei* ATCC 6258. MICs were visually determined after 24 h of incubation at 35 °C, following CLSI recommendations [18]. For fluconazole and the tested echinocandins, the partial-inhibition endpoint (≥ 50% reduction in growth compared with the positive control) was applied, in accordance with CLSI guidelines. For amphotericin B, the complete inhibition criterion (100% growth reduction relative to the positive control) was used, as recommended for polyene antifungals.

### Statistical Analysis

All statistical analyses were performed using R (version 4.3.0). Continuous variables were summarised as medians with interquartile ranges, while categorical variables were expressed as counts and percentages. Variables originally recorded as binary integers (0/1) were recorded as categorical factors labeled as “No” and “Yes”. Clinical outcome was dichotomised as favourable (0) or death (1). Associations between categorical variables were assessed using Fisher’s exact test, given the small sample size and the presence of sparse cell counts. Differences in length of hospital stay between two groups were evaluated using the Wilcoxon rank–sum test, whereas comparisons across multiple infection sites were performed using the Kruskal–Wallis test. To identify independent predictors of mortality, a Firth penalised logistic regression model was fitted using the logistfpackage, which provides bias-reduced estimates suitable for rare events and small sample sizes. To minimise the risk of overfitting, the multivariable model included three clinically relevant covariates: age (continuous), ICU stay (yes/no), and bloodstream infection (yes/no). Odds ratios (ORs) with 95% confidence intervals (CIs) were derived by exponentiating the penalised regression coefficients and the corresponding profile penalised likelihood bounds. All statistical tests were two-sided, and *p*-values < 0.05 were considered statistically significant.

## Results

Between January 1, 2019 and December 31, 2025, *S. cerevisiae* was identified from samples of 196 different patients, of which 30 cases (15%) represented isolates obtained from sterile body sites. A total of 30 patients with sterile-site *S. cerevisiae* infections were analyzed. The cohort consisted of 16 females and 14 males, with a mean age of 47.2 years (range: 0–84 years). Notably, seven patients (23%) were under 18 years of age and categorized as pediatric cases. Bloodstream infections were the most common source (n = 7, 23%), followed by intra-abdominal abscess aspirates and drained intra-abdominal wounds (each n = 5, 17%), and intra-operative colon wound samples (n = 4, 13%). Drained pleural fluid and drained wound exudate accounted for two samples each (7%), while all other sources (burn wound, lung abscess aspirate, drained peritoneal fluid, retroperitoneal abscess aspirate, drained colon abscess) were rare (each n = 1, 3%). Concomitant bacterial pathogens were identified in 21/30 cases (70%), as detailed in Table 1, most commonly *Acinetobacter* spp., *Klebsiella pneumoniae*, *Enterococcus* spp., and *Pseudomonas aeruginosa*. However, in 9 cases (30%), *S. cerevisiae* was the sole pathogen. Peritonitis was the most common underlying condition, affecting 23% of patients (n = 7), and solid tumors were found in an equal proportion of cases (23%, n = 7). Prior gastrointestinal surgery was documented in 63% of patients (n = 19). Hospitalization was often prolonged, with a median length of stay of 23.5 days (IQR: 8.25–50.75 days). In addition, ten patients (33%) required admission to the ICU, and nine patients (30%) had a history of *S. ‘boulardii’* probiotic exposure (Table 1).

**Table 1:** Demographic and clinical characteristics, risk factors, and outcomes of patients with *Saccharomyces cerevisiae* isolation

Age/Gender	Site of isolation	Polymicrobial	Underlying condition	ICU^1 ^stay	Severe neutropenia	Central line	Parenteral nutrition	Treatment	Length of stay (days)	Diabetes	Outcome	*S. ‘boulardii’* therapy	GI^2^ surgery
67/Female	Burn wound	A. bau, K. pneu, E. fae	Rheumatoid arthritis	No	Yes	Yes	Yes	Anidulafungin	41	No	Favourable	Yes	No
84/Female	Drained intra-abdominal wound	A. pit, P. aer	Peritonitis	Yes	No	Yes	Yes	Fluconazole	8	No	Death	No	Yes
32/Female	Drained intra-abdominal wound	E. fcm	Peritonitis	No	No	No	No	Fluconazole	9	No	Favourable	No	Yes
65/Male	Blood	No	GI ulcer	No	No	No	No	Fluconazole	17	Yes	Favourable	No	No
24/Male	Intra-abdominal abscess aspirate	S. ang	Peritonitis	No	No	No	Yes	Fluconazole	12	No	Favourable	No	Yes
67/Female	Blood	E. coli	Peritonitis	Yes	No	Yes	Yes	NA^3^	6	No	Death	Yes	Yes
16/Male	Intra-operative colon wound sample	K. pneu, E. coli, S. aga	GI ulcer	No	No	No	No	NA^3^	6	No	Favourable	No	No
68/Female	Intra-abdominal abscess aspirate	P. aer, S. mal, E. fae, E. coli	Solid malignancy	Yes	No	Yes	Yes	Fluconazole	10	No	Death	No	Yes
8/Female	Drained intra-abdominal wound	P. aer, K. pneu, E. fae	Solid malignancy	No	Yes	Yes	Yes	Fluconazole	226	No	Favourable	Yes	Yes
74/Male	Drained intra-abdominal wound	P. aer	Solid malignancy	Yes	No	Yes	Yes	NA^3^	24	No	Death	No	Yes
54/Female	Drained peritoneal fluid	No	Solid malignancy	No	No	No	No	NA^3^	27	No	Favourable	No	Yes
50/Female	Intra-operative colon wound sample	S. con	GI ulcer	Yes	No	Yes	Yes	Fluconazole	5	No	Favourable	No	Yes
61/Female	Intra-abdominal abscess aspirate	No	Solid malignancy	No	No	Yes	Yes	Anidulafungin	37	No	Death	No	Yes
48/Male	Retroperitoneal abscess aspirate	E. fcm	Pancreatitis	No	No	No	Yes	NA^3^	61	Yes	Favourable	No	Yes
64/Male	Intra-operative colon wound sample	E. fae	GI ulcer	No	No	No	No	NA^3^	8	No	Favourable	No	Yes
62/Male	Lung abscess aspirate	S. aur, A. bau, E. fae, P. aer	Pneumonia	No	No	No	No	NA^3^	54	No	Favourable	No	No
72/Female	Blood	No	Kidney failure	Yes	No	Yes	Yes	Anidulafungin	56	No	Death	Yes	No
66/Male	Drained pleural fluid	A. bau, E. fcm, S. mal, P. aer	Pneumonia	Yes	No	Yes	Yes	Anidulafungin	23	No	Death	No	No
48/Female	Blood	No	Ileus	Yes	No	Yes	Yes	Anidulafungin	17	No	Favourable	Yes	Yes
0/Male	Blood	No	Gastroenteritis	No	No	No	No	Fluconazole	4	No	Favourable	Yes	No
66/Male	Drained intra-abdominal wound	B. fra	Solid malignancy	No	No	No	No	Anidulafungin	8	No	Favourable	Yes	Yes
2/Male	Drained pleural fluid	No	Peritonitis	Yes	No	Yes	Yes	Fluconazole	184	No	Favourable	No	No
6/Female	Drained wound exudate	P. oris	Pneumonia	No	No	No	No	Fluconazole	67	No	Favourable	No	No
78/Male	Blood	No	Gastroenteritis	No	No	Yes	Yes	Fluconazole	99	No	Favourable	No	Yes
59/Male	Drained colon abscess	E. fae, E. fcm	Solid malignancy	No	No	Yes	No	NA^3^	29	No	Death	No	Yes
56/Female	Intra-abdominal abscess aspirate	C. ter, E. coli, K. pneu	Peritonitis	No	No	No	No	NA^3^	66	No	Favourable	Yes	Yes
13/Female	Intra-abdominal abscess aspirate	Ent. hor	Peritonitis	No	No	No	No	Fluconazole	24	No	Favourable	Yes	Yes
16/Female	Intra-operative colon wound sample	E. coli	GI ulcer	No	No	No	No	NA^3^	6	No	Favourable	No	Yes
38/Male	Drained wound exudate	E. fae, P. mi	Soft tissue infection	No	No	No	No	NA^3^	11	Yes	Favourable	No	No
53/Female	Blood	No	Sepsis	Yes	No	Yes	No	Anidulafungin	30	No	Favourable	No	No

A. bau, *Acinetobacter baumannii*; A. pit, *Acinetobacter pittii*; E. fae, *Enterococcus faecalis*; E. fcm, *Enterococcus faecium*; P. aer, *Pseudomonas aeruginosa*; K. pneu, *Klebsiella pneumoniae*; P. mi, *Proteus mirabilis*; E. coli, *Escherichia coli*; S. mal, *Stenotrophomonas maltophilia*; S. ang, *Streptococcus anginosus*; S. aga, *Streptococcus agalactiae*; S. con, *Streptococcus constellatus*; Ent. hor, *Enterobacter hormaechei*; C. ter, *Clostridium tertium*; P. oris, *Prevotella oris*; B. fra, *Bacteroides fragilis*; S. aur, *Staphylococcus* aureus

^1^Intensive care unit stay

^2^Gastrointestinal tract surgery

^3^Not applicable

Antifungal therapy was administered to 19 patients, with fluconazole (n = 12, 63%) and anidulafungin (n = 7, 37%) being the most frequently prescribed agents. In contrast, no antifungal treatment was documented in 11 of the 30 cases (37%). Overall, 22 patients (73%) achieved a favourable outcome, whereas mortality occurred in eight cases, corresponding to an all-cause mortality rate of 27%. Mortality analyses revealed several relevant associations. ICU admission was strongly associated with death (Table 1). Patients requiring critical care had a significantly higher risk of mortality compared with non-ICU patients (Fisher’s exact test *p* = 0.007; OR = 11.98, 95% CI 1.49–165). The presence of a central venous catheter also correlated significantly with mortality (*p* = 0.002). No significant differences in mortality were detected with respect to severe neutropenia (*p* = 1.00), polymicrobial versus monomicrobial infection (*p* = 1.00), *S. ‘boulardii’* probiotic exposure (*p* = 0.676), or the presence of bloodstream infection (*p* = 1.00). Length of hospital stay did not differ significantly between survivors and non-survivors (Wilcoxon test *p* = 0.742). Similarly, length of hospital stay did not differ considerably according to ICU admission status (*p* = 0.552) or across different sites of isolation (Kruskal–Wallis *p* = 0.148), suggesting that neither anatomical source of infection nor critical care requirement substantially influenced duration of hospitalisation within this small cohort.

To evaluate independent predictors of mortality, a Firth penalised logistic regression model was fitted using age, ICU stay, and bloodstream infection as covariates. Increasing patient age was independently associated with higher odds of death (adjusted OR = 1.11, 95% CI 1.02–1.37, *p* = 0.008). ICU admission remained a significant predictor after adjustment (adjusted OR = 10.09, 95% CI 1.23–259.6, *p* = 0.03), underscoring the role of critical illness severity in outcomes of sterile-site *Saccharomyces* spp. infections. In contrast, bloodstream infection was not independently associated with mortality in the adjusted model (adjusted OR = 0.27, 95% CI 0.0079–2.56, *p* = 0.273). Finally, although patients exposed to *S. ‘boulardii’* exhibited a numerically higher rate of bloodstream infection (44% vs. 14%), this difference did not reach statistical significance (Fisher’s exact test *p* = 0.153; OR = 4.8, 95% CI 0.91–22.84).

Based on the available ECVs, all isolates were classified as wild type for micafungin, anidulafungin, and caspofungin (Table 2). In contrast, two isolates were categorized as non–wild type for fluconazole, one of which also exhibited a non–wild type phenotype for amphotericin B. The detailed antifungal susceptibility profile revealed considerable variability. Fluconazole showed a median MIC of 8 µg/mL, with a wide distribution from 0.125 to 128 µg/mL, indicating frequent reduced susceptibility (Table 2). Amphotericin B demonstrated lower MIC values with a median of 0.25 µg/mL (range 0.016–4 µg/mL). Echinocandins retained the greatest potency, with anidulafungin showing a median MIC of 0.008 µg/mL (range 0.008–0.016 µg/mL), caspofungin 0.125 µg/mL (range 0.064–0.25 µg/mL), and micafungin 0.064 µg/mL (range 0.008–0.25 µg/mL) (Table 2). These findings highlight that although echinocandins and amphotericin B generally maintained strong activity against clinical isolates of *S. cerevisiae*, decreased fluconazole efficacy was frequent and may have contributed to the variable clinical outcomes observed.

**Table 2:** Distribution of minimal inhibitory concentrations (MICs) of antifungal agents against clinical *Saccharomyces cerevisiae* isolates (n = 30)

Antifungals	MIC^1^ range (µg/mL)	Median MIC (µg/mL)	MIC_50_ (µg/mL)	MIC_90_ (µg/mL)
Fluconazole	0.125–128	8	4	32
Amphotericin B	0.016–4	0.25	0.25	0.5
Anidulafungin	0.008–0.016	0.008	0.008	0.016
Caspofungin	0.064–0.25	0.125	0.125	0.25
Micafungin	0.008–0.25	0.064	0.064	0.064

^1^ MIC: Minimal Inhibitory Concentration

## Discussion

Although sterile-site infections caused by *S. cerevisiae* remain rare, published data over the past two decades indicate an increasing recognition of such cases, particularly in association with probiotic use [5, 20–24]. Sterile-site disease caused by *S. cerevisiae* is thought to occur via two main routes, namely translocation from the gastrointestinal lumen or breaches in the skin barrier, primarily in the context of intravascular devices [5]. During the seven-year study period, a total of 196 clinical cases with isolation of *S. cerevisiae* were identified at our institution. Of these, 30 cases (15%) fulfilled the predefined criteria for sterile-site infection. Although the number of cases presented may appear high compared with earlier case series, our institution is a large tertiary-care centre with extensive surgical and intensive care activity, and we included sterile-site infections from multiple normally sterile anatomical sites rather than bloodstream infections alone. Importantly, the reported 15% reflects the proportion of sterile-site cases among all patients with *S. cerevisiae* isolation at our clinical centre and should not be interpreted as a population-based incidence. This proportion is consistent with previously published reports and further supports the recognition of *S. cerevisiae* as an emerging opportunistic fungal pathogen. Our findings are in line with recommendations issued by the ECMM, in collaboration with the ISHAM and the ASM, which emphasize the clinical relevance of invasive infections caused by this microorganism [7, 8].

The demographic and clinical characteristics of our population are largely consistent with those reported in previously published epidemiological studies [4, 5, 8, 12, 26, 28]. The study cohort predominantly consisted of middle-aged adults, with a substantial proportion of paediatric patients, and was characterized by high rates of gastrointestinal surgical interventions, ICU admission, and central venous catheter use. The gastrointestinal tract is considered a major reservoir from which *S. cerevisiae* can translocate due to mucosal barrier disruption, abdominal surgical intervention, or dysbiosis [5]. This pathophysiological mechanism is supported by both experimental and clinical evidence, including epidemiological studies demonstrating genetic identity between endogenous gastrointestinal strains and bloodstream isolates [4]. In our study, the high proportion of patients with prior gastrointestinal surgery further supports the strong association between abdominal interventions and sterile-site *S. cerevisiae* infections.

A substantial proportion of the available literature on invasive *Saccharomyces* spp. infections focuses on fungemia; and these data constitute the primary reference framework [8, 12, 13, 20, 25–27]. In a comprehensive review, Muñoz et al. reported that 60% of patients with *Saccharomyces* spp. fungaemia were admitted to the ICU, and the majority received total parenteral nutrition [26]. In our examined population, ICU admission accounted for 33% of all sterile-site cases; however, when fungaemic episodes were analyzed separately, ICU involvement was observed in 71% of cases. Similarly, total parenteral nutrition was administered in 50% of our patients.

A recent systematic review analysing 108 cases of *Saccharomyces* spp. bloodstream infection reported that 68% occurred in patients who had received *S. cerevisiae* var. *‘boulardii’* probiotic therapy [8]. The most frequently identified risk factors included ICU admission (32%), total parenteral nutrition (30%), gastrointestinal symptoms (21%), and diabetes mellitus (13%). Prior use of the *S. ‘boulardii’* probiotic shows a strong association with the development of fungemia. In a case–control study, patients with *S. cerevisiae* fungaemia had a markedly increased odds of prior probiotic use (OR 14, 95% CI 4–44) compared with patients with bacteraemia or candidaemia [21]. This Finnish nationwide 10-year survey showed 46 cases of *Saccharomyces* spp. fungemia, of which 43% occurred in patients receiving probiotics [21]. Poncelet et al. described ten cases of *S. cerevisiae* fungaemia, the majority of which were associated with probiotic formulations containing *S. cerevisiae var. ‘boulardii’* [20]. Similarly, Roy et al*.* reported seven cases from two tertiary-care hospitals, with molecular confirmation of clonal identity between blood isolates and the administered probiotic strains [12]. A large retrospective analysis from the United States identified fungemia in 18 probiotic recipients, and a further systematic review confirmed probiotic exposure in 68% of reported cases worldwide [27]. In patients without prior probiotic use, immunosuppression, gastrointestinal surgery, and intravenous drug usage were the most frequently identified predisposing factors [27]. In our study, patients exposed to probiotics exhibited a numerically higher rate of bloodstream infection compared with non-exposed patients (44% vs. 14%); however, this difference did not reach statistical significance, most likely due to the limited sample size and consequently low statistical power. It is noteworthy that literature reviews have shown 40–87% of *Saccharomyces* spp. fungaemia cases are associated with prior probiotic administration. Importantly, invasive *S. cerevisiae* infection may also occur in transplant recipients in the absence of documented probiotic ingestion, most commonly through disruption of the gastrointestinal tract barrier or via vascular catheters [4, 28].

Clinical outcomes in sterile-site *Saccharomyces* spp. infections appear to be driven predominantly by host-related factors rather than pathogen-intrinsic characteristics. Poncelet et al. reported that among the patients, 80% required total parenteral or enteral nutrition, 70% had a central venous catheter, and 30% were in an ICU [20]. Similar conclusions were drawn by Enache-Angoulvant and Hennequin in their comprehensive review [5]. Among 84 cases with available outcome data, only 69% achieved a favourable recovery, indicating that approximately one-third of patients either died or failed to improve. Notably, mortality rates did not differ significantly between immunocompromised and immunocompetent patients, suggesting that adverse outcomes are more strongly associated with cumulative critical illness factors – such as the presence of central venous catheters, severe gastrointestinal disease, or ICU admission – than with immune status alone [5]. Consistent with these observations, the all-cause 30-day mortality rate in our cohort was 27%. Multivariable analysis identified increasing age and ICU admission as independent predictors of mortality. The overall model fit was statistically significant (likelihood ratio test *p* = 0.002), suggesting that the selected predictors explained part of the variability in clinical outcomes despite the small sample size. Furthermore, the strong association between central venous catheter presence and fatal outcome in our cohort—where all fatal cases occurred in catheterized patients—supports existing evidence implicating catheter usage as a major pathogenic and prognostic determinant in sterile-site *Saccharomyces* spp. disease. This is further corroborated by reports documenting a measurable incidence of probiotic-associated *S. ‘boulardii’* bloodstream infection of 0.26 cases per 1,000 central-line days [27]. Collectively, these findings underscore the central role of intravascular devices in both the emergence and outcome of invasive *Saccharomyces* spp. disease [20].

In vitro antifungal susceptibility testing at our institution revealed substantial variability in fluconazole MICs, ranging from 0.125 to 128 µg/mL, with a median MIC of 8 µg/mL, indicating reduced or heterogeneous susceptibility. This pattern is consistent with prior studies describing elevated azole MICs and variable susceptibility profiles among *S. cerevisiae* isolates [14, 29–31]. In vitro susceptibility testing showed low MIC values for amphotericin B and echinocandins, whereas fluconazole MICs were more variable consistent with observations from global surveillance studies and current guideline recommendations. Current ECMM guidance supports amphotericin B as first-line therapy and echinocandins as a first-line alternative for severe invasive *Saccharomyces* spp. infections, reflecting the variable activity of azoles and the absence of standardized clinical breakpoints. [7, 14, 32]. Nearly one-third of patients in our cohort did not receive antifungal therapy, most commonly due to clinical improvement following source control alone. When administered, treatment consisted primarily of fluconazole or anidulafungin. Antifungal selection in this retrospective cohort reflected real clinical decision-making and may have been influenced by concerns regarding amphotericin B toxicity, patient comorbidities, renal impairment, and perceived response to source control, rather than strict adherence to guideline-preferred therapy. Although successful outcomes have been reported with various antifungal agents, effective management is generally attributed to a multifaceted approach combining antifungal therapy, removal of intravascular devices, and discontinuation of probiotic administration. Our findings further support this strategy, particularly in critically ill patients. Overall, our data are in line with current ECMM recommendations by confirming the predominance of gastrointestinal surgery and central venous catheter use as major risk factors and the favourable in vitro activity of echinocandins and amphotericin B against *S. cerevisiae* isolates [7, 8].

Despite addressing an important knowledge gap, this study has several limitations. It is a retrospective, single-centre study, which restricts causal inference and generalisability. The small number of sterile-site *S. cerevisiae* cases reflects the rarity of the condition and limited statistical power, despite the use of bias-reduced regression methods. Moreover, microbiological data were derived from routine clinical diagnostics, and systematic molecular typing was not available for all isolates; therefore, definitive strain-level linkage between probiotic formulations and clinical isolates could not be performed routinely. It is noteworthy that cases were classified based on isolation from specimens considered sterile sites; however, direct microscopy or histopathological confirmation was not available for all non-blood samples. Consequently, especially in deep wound or intra-abdominal specimens obtained after gastrointestinal surgery with concomitant bacterial species, some episodes may have represented colonisation rather than true infection, potentially leading to an overestimation of clinically significant cases. Further well-designed cohort studies are therefore needed to clarify the real etiological role of *S. cerevisiae* in these clinical settings.

In conclusion, this study expands the currently limited institutional data on sterile-site *Saccharomyces* spp. infections by providing a multi-year evaluation of epidemiological, clinical, and microbiological characteristics in a large Central European tertiary-care hospital. Our findings indicate that the epidemiological burden of such infections among hospital isolates is non-negligible. Future research should therefore focus on establishing clinical breakpoints, refining therapeutic recommendations, and developing risk stratification tools to better identify patients at risk of serious disease, particularly in the context of probiotic exposure.

## Data Availability

The data that support the findings of this study are available from the corresponding authors upon reasonable request.
